# Molecular regulators of defective placental and cardiovascular development in fetal growth restriction

**DOI:** 10.1042/CS20220428

**Published:** 2024-06-21

**Authors:** Anandita Umapathy, Alys Clark, Arvind Sehgal, Vijaya Karanam, Gayathri Rajaraman, Bill Kalionis, Helen N. Jones, Jo James, Padma Murthi

**Affiliations:** 1 Department of Obstetrics and Gynaecology, Faculty of Medical and Health Sciences, University of Auckland, New Zealand; 2 Auckland Bioengineering Institute, Bioengineering Institute, New Zealand; 3 Department of Paediatrics, Monash University, Melbourne, VIC, Australia and Monash Newborn, Monash Children’s Hospital, Melbourne, VIC, Australia; 4 Department of Obstetrics, Gynaecology and Newborn Health, University of Melbourne and Royal Women’s Hospital, Victoria, Australia; 5 First year college, Victoria University, St Albans, Victoria 3021, Australia; 6 Department of Maternal Fetal Medicine, Pregnancy Research Centre, Royal Women’s Hospital, Victoria, Australia; 7 Department of Physiology and Aging, University of Florida College of Medicine, Gainesville, FL, U.S.A.; 8 Center for Research in Perinatal Outcomes, University of Florida College of Medicine, Gainesville, FL, U.S.A.; 9 Department of Pharmacology, Biomedicine Discovery Institute, Monash University, Clayton, Victoria, Australia

**Keywords:** angiogenic factors, cardiovascular development, Fetal growth, growth factors, homeobox genes, Placenta

## Abstract

Placental insufficiency is one of the major causes of fetal growth restriction (FGR), a significant pregnancy disorder in which the fetus fails to achieve its full growth potential in utero. As well as the acute consequences of being born too small, affected offspring are at increased risk of cardiovascular disease, diabetes and other chronic diseases in later life. The placenta and heart develop concurrently, therefore placental maldevelopment and function in FGR may have profound effect on the growth and differentiation of many organ systems, including the heart. Hence, understanding the key molecular players that are synergistically linked in the development of the placenta and heart is critical. This review highlights the key growth factors, angiogenic molecules and transcription factors that are common causes of defective placental and cardiovascular development.

## Introduction

Fetal growth restriction (FGR) has lifelong impacts on babies, including an increased risk of developing chronic adulthood diseases such as diabetes and cardiovascular disease [1]. *In utero*, the placenta and fetal heart develop concurrently, and as such are functionally linked in terms of both biomechanical parameters and shared developmental signalling molecules/pathways [2]. Increasing evidence suggests that this heart–placenta axis is impaired in FGR, with FGR placentae exhibiting impaired vascular development that could contribute to an increased load on the fetal heart [3]. FGR hearts exhibit signs of cardiac remodelling and impaired myocardial performance that follow a phenotypic spectrum related to FGR severity [4]. Multiple underlying mechanisms underpin dysfunction in the heart–placenta axis, and contribute to the pathological impacts of FGR *in utero* and throughout life. This review aims to integrate understanding of these mechanisms across molecular, cellular, anatomical and functional scales.

## Fetal growth restriction

Fetal growth restriction (less than third growth percentile, as defined by a Dephi consensus) occurs as a result of placental insufficiency that prevents a fetus from reaching its genetically determined growth potential [5]. FGR occurs in 5–8% of term pregnancies, but accounts for 17–30% of preterm deliveries, and is thus associated with increased fetal morbidity and mortality [6,7]. Indeed, FGR is the greatest risk factor for stillbirth [8]. Postnatally, FGR babies have a higher incidence of developing neurodevelopmental disorders, metabolic disorders and cardiovascular disease, which persist into adolescence and adulthood. *In utero* diagnosis of FGR is missed in more than half of cases, meaning that best clinical practice cannot be applied to manage these pregnancies [9]. Even for FGR cases that are diagnosed *in utero*, there is currently no effective treatment. As such, FGR can often result in preterm delivery to reduce fetal morbidity [8].

FGR can be classified as early (<32 weeks) or late (>32 weeks) onset according to the gestation at which it is first diagnosed [5]. Early onset FGR accounts for 20–30% of FGR cases and is associated with poor clinical outcomes and clinical manifestations such as hypertension and preeclampsia compared with late onset FGR (which constitutes 70–80% of FGR cases) [10]. Finally, whilst the primary focus of this review is on the association between cardiovascular disease and placental insufficiency, it is also worth noting that placental dysfunction has distinct mechanistic links to other disorders such as Type 2 diabetes, likely as a result of impaired β-cell development and dysregulation of glucose tolerance during the perinatal period [11].

## Clinical diagnosis and assessment of FGR with Doppler ultrasound

In addition to babies less than third fetal growth centile, in pregnancies where estimated fetal weight is less than tenth centile Doppler ultrasound parameters can clinically distinguish FGR babies (umbilical and uterine artery Doppler pulsatility indices greater than 95th centile for early onset FGR, relationship of umbilical artery Doppler to the middle cerebral artery (MCA) Doppler for late onset FGR) [5].

An absent/reversed end diastolic blood flow in the umbilical arteries is associated with severe fetal deterioration and increased fetal and neonatal morbidity [12], and inclusion of umbilical artery Doppler in monitoring can decrease perinatal mortality in at risk pregnancies [13]. Umbilical artery Doppler can be correlated to placental vascular anatomy [14]. A supplementary metric obtainable from umbilical artery Doppler, the wave reflection coefficient, is also indicative of high haemodynamic impedance or an impedance mismatch, potentially due to terminal villi capillary impedance [15], and this has been verified in animal studies [16,17]. However, a common caveat with umbilical artery Doppler is measurement variability as changes in fetal heart rate or Doppler sampling site location can markedly affect the signal [15].

Uterine artery Doppler reflects the resistance of the utero-placental circulation, and has particular utility in identifying risk of maternal vascular malperfusion [18]. A high pulsatility index in the uterine Doppler waveform suggests problems with the adaption of the uterine vasculature to pregnancy, and is likely related to both trophoblast induced remodelling of the spiral arteries and trophoblast independent remodelling of the upstream radial arteries [19].

Fetal assessment parameters such as the MCA Doppler can determine how changes in blood flow through the placenta impact the fetus. In response to hypoxia as a result of insufficient blood flow, the fetus will redirect blood to the brain termed the ‘brain sparing’ effect, which is seen as a reduction in the pulsatility index of the MCA [20]. However, this effect is not neuroprotective long term, and an abnormal MCA PI is associated with adverse neurological outcomes [21]. Although the MCA Doppler is valuable for identifying adverse outcomes among late-onset FGR pregnancies, and in distinguishing between late-onset FGR and constitutionally small fetuses, its role in predicting FGR is weak and alone it is not currently included in protocols for diagnosis/management of FGR [22]. However, the ratio of MCA Doppler/umbilical artery Doppler provides the cerebroplacental ratio (CPR) [20], which forms part of Delphi consensus definitions of FGR, and this metric is associated with adverse perinatal outcomes, and is an independent predictor of stillbirth in the third trimester [5,22,23].

## Placental dysfunction in FGR

The placenta has a branching villous structure, with each villous surrounded by a bilayer of trophoblast. The outer syncytiotrophoblast in this bilayer mediates exchange of nutrient and oxygen between the maternal and fetal circulations, whilst the underlying cytotrophoblast proliferates to drive villous growth and fuses to form the overlying syncytiotrophoblast. The trophoblast bilayer surrounds a mesenchymal core containing stromal cells, placental macrophages, and fetal blood vessels.

The insufficient nutrient/oxygen transport that hinders fetal growth in FGR is multifactorial. On the maternal side, this can arise from inadequate maternal blood flow to the placenta due to maladaptation of uterine radial or spiral arteries [19,24]. However, key changes in the placenta itself are also evident, including decreased placental volume, deficits in vascular branching, and insufficient nutrient transport across the syncytiotrophoblast [25]. It is thus clear that a number of different placental abnormalities are associated with FGR, resulting in different disease phenotypes. As a result, it can be difficult to separate the cause and consequence of the pathology.

### Alterations in placental architecture in FGR pregnancies

FGR placentae exhibit anatomical changes at both the whole organ and the villous level. Placentae from human pregnancies that go on to develop FGR have a decreased placental volume, diameter, and thickness in the first trimester compared with those from normal pregnancies, and these changes can persist into the second trimester and significantly affect placental nutrient and gas exchange capacity [26–29]. At a cellular level, FGR placentae exhibit significant reductions in cytotrophoblast volume and the proportion of proliferating cytotrophoblasts per villus compared with normal placentae [30]. *In vitro* both primary cytotrophoblasts and cytotrophoblast-derived syncytiotrophoblast from FGR placentae are more susceptible to tumour necrosis factor-α induced apoptosis [31,32]. Together, this suggests that the balance between trophoblast formation and loss is altered in these placentae.

### Nutrient transport in FGR pregnancies

Insufficient nutrient transport is a significant contributor to the pathogenesis of FGR through either decreased maternal blood flow around the villi or changes in syncytiotrophoblast function. The syncytiotrophoblast mediates nutrient transport from the maternal to the fetal circulation through the expression of amino acid transporters, fatty acid transport proteins and glucose transporters [33]. However, changes in the number and activity of nutrient transporters can lead to decreased nutrient transport. For example, there is a 63% decrease in the expression and activity of amino acid transporter System A in the syncytiotrophoblast of FGR placentae compared with normal pregnancies [34]. Furthermore, blood samples collected from FGR fetuses show decreased levels of glucose and amino acids [35]. However, the decrease in glucose levels is not thought to result from changes in placental glucose transporter expression, but rather seems to be a fetal compensatory adaptation to an increase in the transplacental glucose gradient [36].

## Placental vascular development

The placenta’s ability to support the increasing nutrient and oxygen demands of the growing fetus depends on the successful development of the placental vascular network [37,38]. Across gestation, first vasculogenesis, and then angiogenesis, create an extensively branched vascular network to maximise surface area for nutrient exchange. Disturbances in these processes in FGR can lead to abnormal vascular network development [39]. Indeed, during normal placental development the resistance through the utero-placental circuit drops as gestation progresses, whereas this adaptive process is disrupted in FGR pregnancies, negatively impacting oxygen and nutrient supply and fetal growth [40].

Vasculogenesis (the *de novo* development of blood vessels) begins in humans at day 15 post-fertilisation, with the formation of endothelial cell cords in primitive villi, which subsequently elongate via endothelial cell proliferation, and are stabilised via recruitment of pericytes and vascular smooth muscle cells at around day 28 post fertilisation [41,42]. Following vasculogenesis, the network is expanded via angiogenesis, with branching angiogenesis dominating from 6 to 24 weeks of gestation, after which non-branching angiogenesis further elongates existing vessels untill term [43]. Branching angiogenesis is initiated by trophoblast secretion of an array of angiogenic factors such as vascular endothelial growth factor (VEGF), fibroblast growth factor (FGF) and angiopoietins, inducing proliferation and migration of endothelial cells for the formation of new vascular branches and the expansion of the capillary network [44,45]. The resulting placental vasculature exhibits a mix of dichotomous and monopodial branches (parent vessels with a consistent diameter that branch into smaller diameter daughter branches) which ensures delivery and perfusion of blood over a large distance [46]. Subsequently, non-branching angiogenesis results in a twisting of the vessels into capillary loops by the generation of capillaries that exceed the length of the villi in which they are contained [43]. These capillary loops push up against the syncytiotrophoblast to form vasculo-syncytial membranes, which reduce the diffusion distance to permit adequate nutrient and gas exchange [43].

Aberrant changes in utero-placental blood flow and the development of chorionic plate and villous blood vessels within the placenta occur in FGR [47]. At the macro-scale, chorionic plate arteries are significantly fewer and have a smaller number of branches in FGR compared with normal placentae [48]. Corrosion casts of FGR placentae show significantly shorter arterial vessel length density and significantly longer venous vessel length density compared with normal placentae [48,49]. FGR placentae show significant reductions in terminal villous volume and surface area, with thinner and longer vessels in terminal villi compared with normal placentae [50,51]. Resistance arteries from FGR stem villi also exhibited an increase in vessel wall thickness to lumen ratio in comparison with healthy controls [52], and in line with this an increased perivascular cell:endothelial cell ratio in FGR placentae has been reported [53].

The above alterations in vascular architecture likely arise from cell-level dysfunction [54]. Indeed, the higher capillary shear stress conditions predicted in FGR by *in silico* modelling result in more persistent movement of microvascular endothelial cells, in turn favouring vessel elongation over branching [55]. Perivascular placental mesenchymal stromal cells (pMSCs) are also less angiogenic as evidenced both by their inhibition of endothelial tube formation in vitro, and by their ability to impair the capacity of M2 macrophages to promote endotheial tube formation [54]. Finally, FGR pMSCs express higher levels of fibulin-2, which would stiffen vessels, and a significant decrease in hyaluronan synthase-2 (the enzyme that synthesises pro-angiogenic hyaluronan), which would reduce vessel integrity and growth [56]. Together, these vascular perturbations adversely impact nutrient and gas exchange, thus contributing to the pathogenesis of FGR [55].

## Regulation of placental vascular development

Placental angiogenesis is regulated by a combination of pro- and anti-angiogeneic factors, transcription factors, and intracellular signalling pathways. Such regulatory factors are controlled temporally throughout gestation and in turn drive the temporal regulation of vasculogenesis and angiogenesis during pregnancy [57].

### Growth factors

The VEGF family are important regulators of angiogenesis as placental vascular network formation begins via VEGF secretion which causes proliferation of the endothelial cells and growth of the primitive endothelial cell cords. The actions of VEGF are mediated by binding to its specific receptors, vascular endothelial growth factor receptor-1 (VEGFR-1) and vascular endothelial growth factor receptor-2 (VEGFR-2), although binding to VEGFR-2 leads to a more potent functional effect compared with VEGFR-1 [58]. In the human placentae, VEGF is expressed by the syncytiotrophoblast, perivascular/endothelial cells and stromal cells, with its expression highest in the first trimester before decreasing in the latter stages of pregnancy [59–62].

VEGF signalling plays fundamental roles in placental and embryonic development, as animal models of VEGF knockout lead to severe vascular defects and embryonic death at E10.5 [63,64], and VEGFR-1 ablation leads to vasculogenesis and angiogenesis problems resulting in embryonic lethality [65]. However, the data around VEGF expression in FGR placentae are limited and conflicting. Some authors have shown that there is significantly increased *in vitro* expression of VEGF in the villous stroma and endothelial cells of FGR placentae, potentially as an adaptive response to placental malperfusion [44]. Conversely, others have shown no change in VEGF expression in the villous stroma of FGR placentae compared with controls [66].

Placental growth factor (PlGF) is a homologue of VEGF and acts to increase the activity of VEGF by competing for the VEGFR-1 receptor, resulting in greater levels of free VEGF available to bind to the more potent VEGFR-2 receptor as well as mediating direct effects on endothelial cells [67]. In human placenta, PlGF is expressed by the syncytiotrophoblast, cytotrophoblasts and endothelial cells, with levels peaking approximately 25 weeks of gestation [68]. Functionally, PlGF plays a role in non-branching angiogenesis by increasing endothelial cell proliferation, migration, growth and survival [69].

Murine PlGF knockout models exhibit no defects in vascular development or changes in labyrinth vessel architecture compared with controls, and the fetuses survive normally to term [70]. However, overexpression of PlGF leads to an embryonic lethal phenotype due to aberrant labyrinth vascularisation in the placenta leading to FGR and severe angiogenic defects in the fetuses [71]. It has thus been hypothesised that overexpression of PlGF induces anti-angiogenic effects [71]. However, the role of PlGF in impacting vascular development in FGR placentae remains unclear, with some studies reporting increased PlGF [72], whilst others report decreased PlGF expression *in vitro* [73].

Angiogenin is a member of the RISBASE family of ribonucleases, which exhibit both ribonuclease activity and induce cell migration, invasion and the formation of tubular structures [74]. In the placenta, angiogenin is produced by the villous cytotrophoblasts, syncytiotrophoblast, endothelial cells and pMSCs [75,76]. Angiogenin is induced by other angiogenic factors (e.g. VEGF, fibroblast growth factor-1 and 2) and is produced at higher levels in the first trimester than at term [76,77]. Consequently it is considered a potent vasculogenic inducer at the start of pregnancy where it stimulates proliferation and differentiation of endothelial cells [76,78]. Studies using angiogenin inhibitors have shown that angiogenin functions to ensure the growth of the placental vasculature in the latter stages of pregnancy in order to keep up with the exponential growth of the fetus [76,79].

Angiogenin expression is significantly increased in FGR placentae compared with normal term placentae [80]. This increase in angiogenin may be viewed as a compensatory mechanism to increase angiogenesis to overcome placental insufficiency associated with FGR [80]. However, it is difficult to delineate whether the increase in angiogenin is a cause of FGR or an consequence of the existing FGR phenotype.

## The heart–placenta axis

Aberrant regulation of placental vascular development can have far reaching consequences, as the structural and functional changes seen in FGR placentae can influence fetal heart development through a developmental axis termed the heart–placenta axis. Both the fetal heart and placental vascular tree develop simultaneously in early pregnancy and share similar signalling pathways/molecules including the heart- and neural crest derivative-expressed protein 1 (Hand1), peroxisome proliferator activated receptor γ (PPARγ) and mitogen activated protein kinase 1 (Mapk1) [2]. Furthermore, the mechanism of the expansion of ventricular wall in the fetal heart switches from embryonic to placental control following the onset of maternal blood flow to the placenta. These events suggest that biomechanical forces from the placenta can influence heart development [81].

Perturbations in the heart–placenta developmental axis could begin to explain the development of some congenital heart diseases and the cardiac remodelling associated with placental pathologies such as pre-eclampsia and FGR. Indeed, placentae from pregnancies complicated by hypoplastic heart disease show a significant decrease in the number of terminal villi and a reduction in vascular density [82]. Furthermore, fetal heart defects in animal models seem to occur approximately E10 of pregnancy, when organogenesis becomes dependent on placental function [83]. However, whilst there is evidence that the heart–placenta axis plays an important role in pregnancy, the relationship between the two organs is not yet fully understood. In this section, fetal heart development in humans will be discussed along with the changes observed in the fetal heart in FGR.

### Development of the fetal heart and circulation

Heart development in the human begins at 4 weeks of gestation with the formation of the primitive heart tube by fusion of the first heart field (a temporal and spatial region of cardiac progenitors derived from the anterior lateral mesoderm) [84,85]. Blood flow and contraction in the heart tube leads to peristaltic pumping, which contributes to the ballooning process to form the atrial and ventricular chambers [86]. Shortly afterwards, the primitive heart tube undergoes cardiac looping to establish the left–right asymmetry in the ventricular chambers and the classic morphology associated with a postnatal heart [87]. Abnormalities in the cardiac looping process have been associated with the development of congenital heart defects [87]. By 7 weeks of gestation in humans a primitive 4-chambered heart structure can be seen [88].

Following cardiac looping, the primitive ventricles undergo trabeculation, which is the formation of luminal projections of cardiomyocytes enclosed by the endocardium (trabeculae), and is complete at 8 weeks of gestation in humans [89]. Trabeculae are important as they facilitate increased cardiac output, contractility, conductivity and ensure that the heart can cope with an increase in afterload at birth [89]. Increased cardiomyocyte proliferation in the heart leads to progressive ventricle wall thickening via hypertrophic growth. At term, the fetal heart contains the complete number of cardiomyocytes, and there is very limited capacity for cellular regeneration in the adult heart [90].

The fetal cardiovascular system has structural differences to that of the postnatal cardiovascular system in order to ensure oxygenated blood from the placenta is delivered to the brain and heart whilst bypassing other organs such as, the lungs and liver. These changes include three fetal shunts: the ductus arteriosus, the ductus venosus and the foramen ovale [91].

### The fetal heart in FGR pregnancies

**Figure 1:**
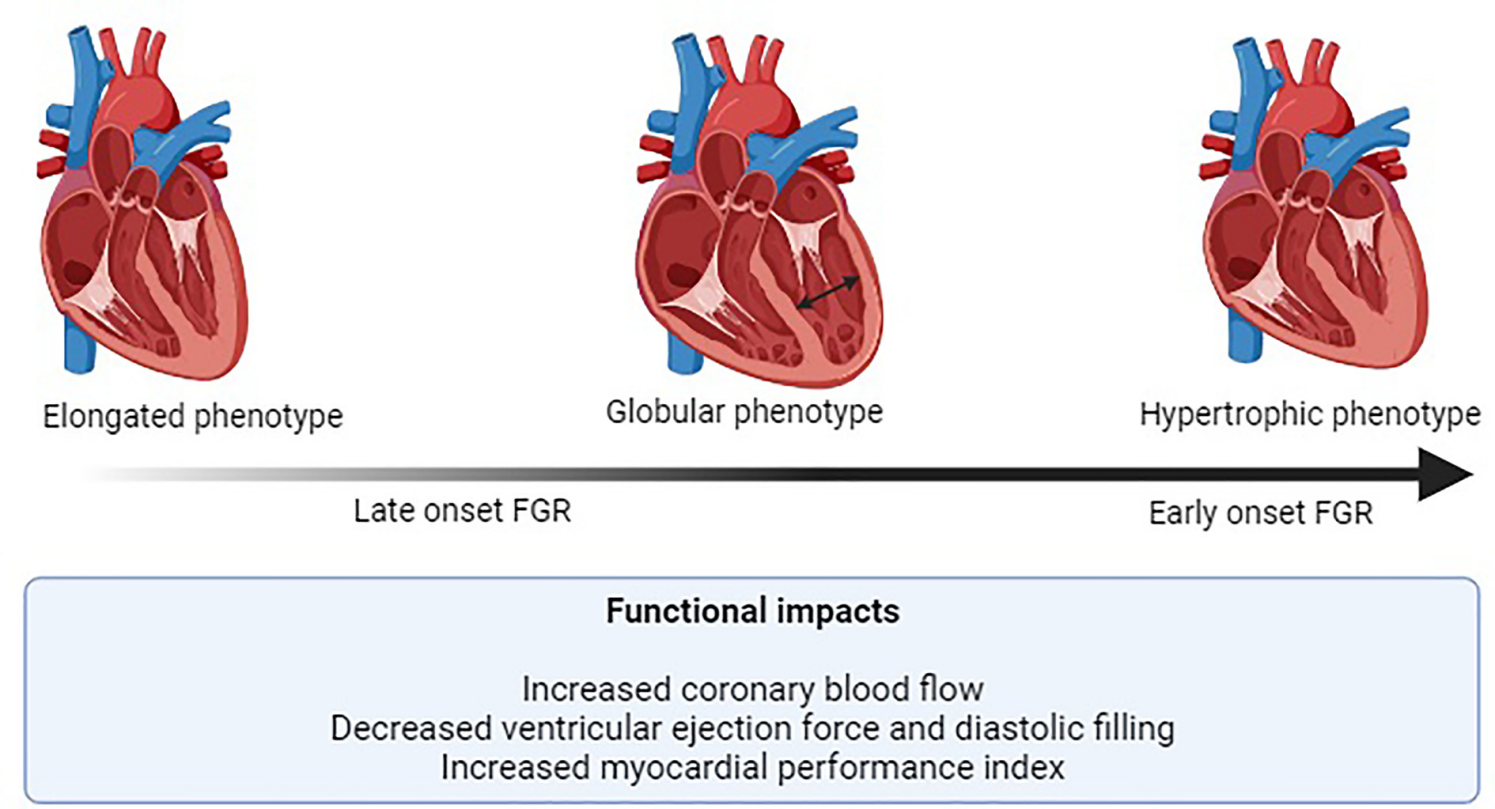
Relationship between placental insufficiency and cardiac remodelling in FGR Changes in growth factor/cytokine expression leads to impaired vascular development in FGR placentae. As a result, these structural changes cause an increase in placental resistance which in turn induces cardiac remodelling in FGR hearts. Cardiac remodelling in FGR exists on a spectrum and is related to FGR severity with severe FGR causing a hypertrophic phenotype (associated with changes in cardiomyocyte volume) while less severe FGR can cause an elongated phenotype however, the microstructure causes for this remodelling phenotype is not known. Together, these changes in cardiac structure lead to impaired heart function. Created with Biorender.

Most organ systems have a limited capacity to handle insults before irreversible structural and functional changes appear, and the fetal heart is no exception. In order to cope with insufficient nutrient and oxygen supply from the placenta, the fetus undertakes ‘brain sparing’ by redirecting blood flow to organs such as the heart and the brain to ensure survival. Indeed, the combined fetal cardiac output fraction to the placenta is significantly decreased in FGR [92]. In turn, placental insufficiency also has direct effects on the fetal heart; (1) the reduction in nutrient and oxygen supply can disrupt cardiomyocyte growth and fibre architecture predominantly through hypoxic mechanisms [4,93], and (2) an increase in placental resistance leads to chronic cardiac afterload as the fetal heart must work harder and generate higher pressures to pump blood through the placenta [94] (Figure 1). Please add the revised image for Figure 1 attached to the email to the publishers - the figure was modified as per reviewers comments. I am unable to insert or upload the revised figure 1 here.

The cardiac remodelling that FGR hearts undergo is described as cardiomyopathy-like, with evidence of dilated ventricles and a change in heart shape occurring in order to maintain ventricular output [95,96]. There are three different heart shapes associated with FGR; (1) the right ventricle becomes globular and pushes the septum leading to an elongated left ventricle (‘elongated phenotype’), (2) both ventricles are elongated (‘globular phenotype’), or (3) in more severe or prolonged cases, the heart undergoes hypertrophy along with a change in shape (‘hypertrophic phenotype’) [4]. Early onset FGR is often associated with the hypertrophic phenotype, whilst late onset FGR is associated with the globular or elongated phenotypes [4] (Figure 1). A globular heart shape allows the heart to better tolerate wall stress by reducing the ventricular radius of curvature and aids in increasing contractile force [97]. Finally, the heart microstructure can be affected in FGR with a significant decrease in the number of cardiomyocytes or a reduction in cardiomyocyte maturation compared with controls [98,99]. This is important as changes to cardiomyocytes can significantly compromise cardiac function.

The structural changes observed in FGR hearts have functional consequences, including a significantly decreased ventricular ejection force and decreased ventricular diastolic filling [100]. This means that the amount of blood pumped to the body is significantly decreased in FGR compared with normal fetuses. Furthermore, the myocardial performance index (MPI), a measure of global cardiac performance, is significantly increased in FGR, and persists in growth restricted infants for up to 3 months of age [101,102]. This is clinically important as an increase in MPI is associated with fetal deterioration and adverse perinatal outcomes [103] (Figure 1). Indeed, increased levels of B-type naturietic peptide and troponin have also been found in the cord blood of both early onset and late onset FGR fetuses in a severity dependent manner, suggesting the heart is experiencing damage [103–105].

Finally, when compared with average for gestational age (AGA) fetuses at preterm, FGR fetuses show earlier visualisation of coronary artery blood flow (CABF) (‘heart sparing’) but impaired diastolic and systolic cardiac function [106] lead to fetal programming for altered cardiac architecture, and/or an effort to maintain myocardial metabolism. Indeed, there is evidence from sheep data that FGR fetuses have selective adaptation to vascular resistance in myocardial vascular beds induced by chronic fetal hypoxaemia [107].

Persistent cardiac remodelling (both structural and functional mal-adaptation) are noticeable during early postnatal period. Recent data in both preterm and term neonates (in comparison with comparable gestation preterm and term infants, respectively) has noted thickened myocardiaum and diastolic and systolic function impairments [106,108]. The systemic arterial circulation is also affected across age groups (increased thickness and stiffness of the aorta and carotid arteries), providing putative links between FGR and adult-onset hypertension [95,108–110].

## Interacting regulators of the heart–placenta axis and their role in FGR pregnancies

Defects in the development of the cardiovascular system have been reported to often co-occur with placental maldevelopment. Experimental approaches in murine models using targeted deletion of specific genes that are only expressed in the placenta have provided evidence that placental insufficiency leads to cardiac defects [111–113]. A more recent study by Radford et al (2023) using novel genetic tools in the generation of embryo- and trophoblast-specific conditional knockouts reported defects in the syncytiotrophoblast layer as a shared and major cause for placental-induced heart defects [114]. However, the molecular mechanisms that underpin the global impact of the placenta on developmental heart disorders remain unknown. It is not surprising to note that there are shared genes and regulatory pathways that control parallel developmental pathways in the placenta and the cardiovascular system, as they are the first organ systems to be formed during mammalian embryogenesis [2]. In this section, we summarise key shared molecular pathways that are critical for the development of both the placenta and fetal heart.

### Common regulatory pathways in the development of placenta and fetal heart

Mechanisms of placental and cardiovascular development share common regulatory pathways. For example, during placental development the precise molecular signaling required for cytotrophoblast fusion into syncytiotrophoblast, and the invasion of extravillous trophoblasts into the decidua are heavily dependent upon the expression of the wingless-related integration site, Wnt proteins. These are secreted proteins that can act as signaling molecules to initiate a variety of intracellular signaling pathways and play a critical role in cell fate decisions, axis patterning, cell proliferation and cellular migration. Wnt signaling is necessary in the developing fetal heart, for myocardial specification, morphogenesis, formation of valves as well as for the proliferation of endothelial cells and vascular smooth muscle cells [115].

Several signal transduction pathways goverened by the growth factor bound receptors such as fibroblast growth factor-receptor (FGFR), epidermal growth factor-receptor (EGFR), bone morphogenic protein (BMP), Notch, Hedgehog, Slit/Robo signaling molecules, including Wnt signaling pathways culminate in the induction of key common transcription factors that are important for the development of placenta and fetal heart [116]. In the following section, two key transcription factors (Hand1 and homeobox genes) that are essential for the development of the fetal heart and placenta are described.

### Hand1

Hand1 is one of the crucial signalling factors in the development of both the fetal heart and placenta. Indeed, a conditional mutation in the Hand1 gene leads to an FGR phenotype in mice, which is characterised by inadequate placental vascular development and decreased apical wall thickness in the fetal heart [117]. The Hand1 gene, which encodes a basic Helix-Loop-Helix (bHLH) transcription factor, regulates downstream target genes that are critical for the development of many organ systems during embryogenesis. In the human placenta, Hand1 mRNA is not detectable by Northern blot in human placental tissue in either the first trimester or at term, although it is thought to be involved in normal trophoblast development as it is expressed in the cell lines Jeg-3 and Be-Wo [118]. Single cell sequencing data further highlights the lack of Hand1 expression in the human placenta [119]. In contrast, Hand1 is abundantly expressed in mouse placenta. At E8.5, Hand1 is expressed in the ectoplacental cone and in primary and secondary trophoblast giant cells (TGCs). At day E14.5, Hand1 expression is found in the chorion, amnion, endothelium and smooth muscle of the umbilical vein and a heterogenous cell population in the murine labyrinth [120].

Besides the placenta, Hand1 is expressed in human and murine hearts, with higher expression in fetal hearts compared with adult hearts [121]. Indeed, mutations in the Hand1 gene have been implicated in the development of certain congenital heart defects and in heart failure [122]. In the murine heart, Hand1 is first detected at E8.5, where it is abundantly expressed in the left ventricle throughout gestation and in the postnatal mouse heart [120]. Hand1 lineage cells give rise to epicardial precursors (coronary smooth muscle cells and cardiac fibroblasts) at E9.5 [120]. Similar to the mouse, Hand1 expression is seen in the first heart field cells in the developing human heart that will give rise to the left ventricle and part of the atria [123].

The importance of Hand1 in placental and heart development is highlighted by the embryonic lethal phenotypes seen in Hand1 knockout animal models. A systemic knockout of Hand1 results in severe extraembryonic and vascular defects and embryonic lethality by E8.5 [124]. Hand1 homozygous null embryos show growth retardation, and by E10.5, embryos showed signs of resorption [125]. Placentae from these embryos showed a reduction in the ectoplacental cone which suggests a dysfunction in the proliferation or maintenance of trophoblast giant cells [125]. Furthermore, Hand1 null embryos arrest just as cardiac development begins, and as the heart is not essential for murine embryo viability until later in gestation this suggests that the arrest in cardiac development was due to extraembryonic defects [125].

### Homeobox genes

Homeobox genes are an important family of growth control genes that encode a highly conserved DNA binding motif; the homeodomain. The specificity of the homeodomain allows these transcription factors/growth control genes to bind to promoter regions of an array of target genes and thereby regulate their expression. Homeobox genes are organised as Hox gene clusters in four distinct chromosomes in mammals. Homeobox genes that are located outside of these clusters are called orphan Hox genes.

We have previously reported in vitro expression of several homeobox genes (*HLX*, *DLX3*, *DLX4*, *DLX5*, *ESX1L*, TALE family homeobox genes, *TGIF* and *MEIS2*) in villous and extravillous trophoblasts, in micro/macrovascular endothelial cells of the placenta and in the mesenchymal stromal cells of the chorionic villi [126–133]. Targeted deletion of Hox genes in murine models have provided evidence for the role of Hox genes in cardiovascular development. More specifically, studies using germline ablation of placental Hox genes, in which the specific Hox gene is absent only in the placenta, have provided the direct evidence for a regulatory role of placental Hox genes in cardiovascular development [134]. For example, the significant role of Hoxa13 in the placenta and heart was demonstrated using a mouse knockout model for Hoxa13 [134]. Hoxa13 was reported to be abundanty expressed in the allantoic bud mesoderm, an earliest developing component of the placenta. However, Hoxa13 was absent in the cardiac crescent, which represents the earliest stages of heart development [135]. In *Hoxa13* knockout mice, significant developmental defects in the heart (thinning of the ventricular wall) was observed secondary to compromised labyrinth vessel branching [135].

**Figure 2:**
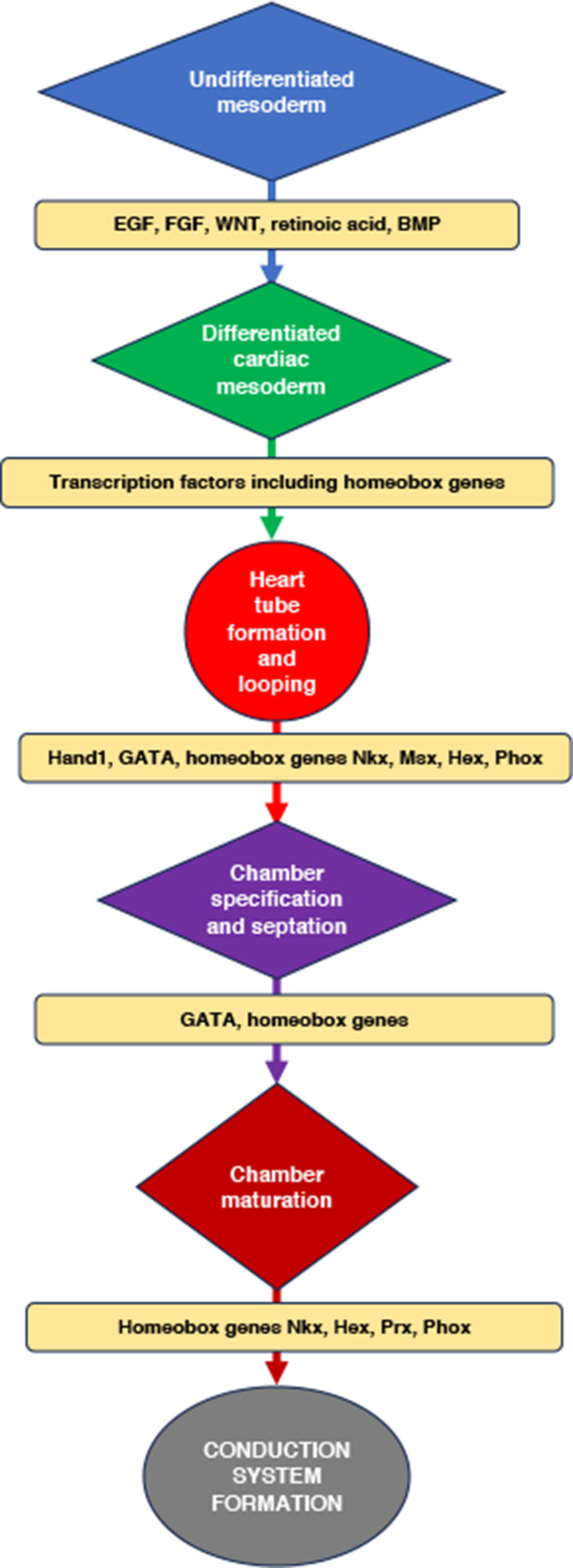
Homeobox genes: their expression and regulation on fetal heart development The expression and regulation of homeobox genes are essential for both placental and fetal heart development. Figure 2 highlights the the cooperative regulation of placental and heart development by homeobox genes. Studies from our own laboratory and from others [125–135] have previously reported that homeobox genes play a crucial role in human placental development. As depicted in the figure, several signal transduction pathways governed by FGF, EGF, Wnt, BMP and retinoic acid have been implicated in the coordination and the establishment of the ordered domains of homeobox gene expression that are essential for fetal heart development including, cardiac cell maturation and in the formation of the functional cardiac conduction system during fetal development. Specifically, homeobox gene, HEX expression is implicated in cardiac mesoderm specifications, while Nkx2-5 expression is essential for heart looping, heart septation and cardiac conduction system formation by integrating BMP, notch and WNT signaling molecules during development (as reviewed by [138].

The emerging roles of several homeobox genes in various aspects of cardiac mesoderm differentiation have been reported and is as depicted in Figure 2. As shown in Figure 2, several signal transduction pathways that are governed by the growth factors, FGF, EGF, Wnt, BMP and retinoic acid may play an important role in the establishment of the ordered domains of homeobox gene expression such as NKX and TALE family homeobox genes that are essential for cardiac maturation and functional cardiac conduction system formation [135, 136]. Our studies using the human placentae have previously reported the expression and localisation of several homeobox genes, including the TALE family homeobox genes *TGIF* and *MEIS* and their potential contribution to placental angiogenesis in FGR and normal pregnancies [126–133]. These homeobox genes are implicated in contributing to patterning of cardiac progenitor cells [137], thus highlighting the importance of potential cooperative regulation of placental and heart development by homeobox genes. Our findings on placental homeobox genes in normal and FGR pregnancies are highly relevant as they may provide novel mechanistic insights into fetal cardiovascular development and diseases.

## Conclusion

The structural, molecular and functional links in the parallel development of the placenta and embryonic heart highlight that inadequate placental vascular development can have a major impacts on the development of the embryonic heart. Extraembryonic development of the placenta may contribute significantly to the increased risk of growth restricted babies in developing cardiovascular disease later on in life. Given the parallel development of the placenta and the heart, and the placenta–heart genetic axis, there is considerable potential for contributions of primary defects from the placenta to have a major impact on pregnancy outcomes. However, the precise molecular events whereby placental development influences normal heart formation, and how this mechanism is disrupted in placental pathologies such as FGR, remains to be elucidated. Improved understanding of this mechanism will enable the development of detection, monitoring and management of impaired cardiac function in FGR babies.

## Data Availability

Not applicable

## Open Access

Open access for this article was enabled by the participation of Monash University in an all-inclusive *Read & Publish* agreement with Portland Press and the Biochemical Society under a transformative agreement with CAUL.

## Abbreviations

AGA: average for gestational age
CABF: coronary artery blood flow
CPR: cerebroplacental ratio
FGR: fetal growth restriction
Hand1: heart- and neural crest derivative-expressed protein 1
Mapk1: mitogen activated protein kinase 1
MCA: middle cerebral artery
PPARγ: peroxisome proliferator activated receptor γ
TGC: trophoblast giant cell
VEGFR: vascular endothelial growth factor receptor
BMP: Bone morphogenic protein
EGF: Epidermal growth factor
FGF: Fibroblast growth factor
WNT: Wingless and Int-1
HLX: H2.0-like homeobox
DLX: Distal-less homeobox
HEX: Hematopoietically expressed homeobox
TALE: Three amino acid loop extension homeobox
TGIF: Transforming Growth Factor B induced factor homeobox
MEIS: Myeloid Ecotropic Viral Integration Site homeobox
Prx: The paired-related homeobox gene
Phox: Paired-like homeobox
NKX: NK family of homeobox

## References

[1] Barker D.J., Eriksson J.G., Forsén T. and Osmond C. (2002) Fetal origins of adult disease: strength of effects and biological basis. Int. J. Epidemiol. 31, 1235–1239 10.1093/ije/31.6.1235 12540728

[2] Wilson R.L., Yuan V., Courtney J.A., Tipler A., Cnota J.F. and Jones H.N. (2022) Analysis of commonly expressed genes between first trimester fetal heart and placenta cell types in the context of congenital heart disease. Sci. Rep. 12, 1–9 10.1038/s41598-022-14955-8 35750800 PMC9232495

[3] Crispi F., Sepúlveda-Martínez Á., Crovetto F., Gómez O., Bijnens B. and Gratacós E. (2020) Main patterns of fetal cardiac remodeling. Fetal Diagn. Ther. 47, 337–344 10.1159/000506047 32213773

[4] Rodríguez‐López M., Cruz‐Lemini M., Valenzuela‐Alcaraz B., Garcia‐Otero L., Sitges M., Bijnens B. et al. (2017) Descriptive analysis of different phenotypes of cardiac remodeling in fetal growth restriction. Ultrasound Obstet. Gynecol. 50, 207–214 10.1002/uog.17365 27859818

[5] Gordijn S.J., Beune I.M., Thilaganathan B., Papageorghiou A., Baschat A.A., Baker P.N. et al. (2016) Consensus definition of fetal growth restriction: a Delphi procedure. Ultrasound Obstet. Gynecol. 48, 333–339 10.1002/uog.15884 26909664

[6] Arroyo J.A. and Winn V.D. (2008) Vasculogenesis and angiogenesis in the IUGR placenta. Semin. Perinatol. 32, 172–177 10.1053/j.semperi.2008.02.006 18482617

[7] Sehgal K., Sehgal K. and Tan K. (2021) Increased incidence of chronic lung disease and respiratory sequelae in growth restricted versus appropriately grown preterms. Indian J. Child Health 8 8 280–283 10.32677/ijch.v8i8.2979

[8] Gardosi J., Madurasinghe V., Williams M., Malik A. and Francis A. (2013) Maternal and fetal risk factors for stillbirth: population based study. BMJ 346, f108 10.1136/bmj.f108 23349424 PMC3554866

[9] Roex A., Nikpoor P., van Eerd E., Hodyl N. and Dekker G. (2012) Serial plotting on customised fundal height charts results in doubling of the antenatal detection of small for gestational age fetuses in nulliparous women. Aust. N. Z. J. Obstet. Gynaecol. 52, 78–82 10.1111/j.1479-828X.2011.01408.x 22309365

[10] Unterscheider J., Daly S., Geary M.P., Kennelly M.M., McAuliffe F.M., O'Donoghue K. et al. (2013) Optimizing the definition of intrauterine growth restriction: the multicenter prospective PORTO study. Obstet. Gynecol. 208, 290.e1–290.e6 10.1016/j.ajog.2013.02.007 23531326

[11] Boehmer B.H., Limesand S.W. and Rozance P.J. (2017) The impact of IUGR on pancreatic islet development and β-cell function. J. Endocrinol. 235, R63–R76 10.1530/JOE-17-0076 28808079 PMC5808569

[12] Caradeux J., Martinez-Portilla R.J., Basuki T.R., Kiserud T. and Figueras F. (2018) Risk of fetal death in growth-restricted fetuses with umbilical and/or ductus venosus absent or reversed end-diastolic velocities before 34 weeks of gestation: a systematic review and meta-analysis. Obstet. Gynecol. 218, S774.e21–S782.e21 10.1016/j.ajog.2017.11.566 29233550

[13] Alfirevic Z., Stampalija T. and Dowswell T. (2017) Fetal and umbilical doppler ultrasound in high‐risk pregnancies. Cochrane Database System. Rev. 13 6 6 10.1002/14651858.CD007529.pub4 PMC6481396 28613398

[14] Sağol S., Sağol Ö. and Özdemir N. (2002) Stereological quantification of placental villus vascularization and its relation to umbilical artery doppler flow in intrauterine growth restriction. Prenat. Diagn. 22, 398–403 10.1002/pd.323 12001195

[15] Cahill L.S., Stortz G., Chandran A.R., Milligan N., Shinar S., Whitehead C.L. et al. (2021) Wave reflections in the umbilical artery measured by doppler ultrasound as a novel predictor of placental pathology. EBioMedicine 67, 103326 10.1016/j.ebiom.2021.103326 33965347 PMC8176120

[16] Cahill L.S., Shinar S., Whitehead C.L., Hobson S.R., Stortz G., Ayyathurai V. et al. (2021) Sex differences in modulation of fetoplacental vascular resistance in growth-restricted mouse fetuses following betamethasone administration: comparisons with human fetuses. Am. J. Obstet. Gynecol. MFM 3, 100251 10.1016/j.ajogmf.2020.100251 33451599 PMC7811575

[17] Cahill L.S., Zhou Y., Hoggarth J., Yu L.X., Rahman A., Stortz G. et al. (2019) Placental vascular abnormalities in the mouse alter umbilical artery wave reflections. Am. J. Physiol. Heart Circ. Physiol. 316, H664–H672 10.1152/ajpheart.00733.2018 30632765 PMC6425519

[18] Agrawal S., Parks W.T., Zeng H.D., Ravichandran A., Ashwal E., Windrim R.C. et al. (2022) Diagnostic utility of serial circulating placental growth factor levels and uterine artery doppler waveforms in diagnosing underlying placental diseases in pregnancies at high risk of placental dysfunction. Obstet. Gynecol. 227, 618.e1–618.e16 10.1016/j.ajog.2022.05.043 35644246

[19] Clark A.R., James J.L., Stevenson G.N. and Collins S.L. (2018) Understanding abnormal uterine artery doppler waveforms: a novel computational model to explore potential causes within the utero-placental vasculature. Placenta 66, 74–81 10.1016/j.placenta.2018.05.001 29884305 PMC6511649

[20] Dall'Asta A., Brunelli V., Prefumo F., Frusca T. and Lees C.C. (2017) Early onset fetal growth restriction. Maternal Health, Neonatol. Perinatol. 3, 1–12 10.1186/s40748-016-0041-x PMC5241928 28116113

[21] Roza S.J., Steegers E.A., Verburg B.O., Jaddoe V.W., Moll H.A., Hofman A. et al. (2008) What is spared by fetal brain-sparing? fetal circulatory redistribution and behavioral problems in the general population Am. J. Epidemiol. 168, 1145–1152 10.1093/aje/kwn233 18826969

[22] Figueras F., Savchev S., Triunfo S., Crovetto F. and Gratacos E. (2015) An integrated model with classification criteria to predict small‐for‐gestational‐age fetuses at risk of adverse perinatal outcome. Ultrasound Obstet. Gynecol. 45, 279–285 10.1002/uog.14714 25358519

[23] Khalil A., Morales‐Roselló J., Townsend R., Morlando M., Papageorghiou A., Bhide A. et al. (2016) Value of third‐trimester cerebroplacental ratio and uterine artery doppler indices as predictors of stillbirth and perinatal loss. Ultrasound Obstet. Gynecol. 47, 74–80 10.1002/uog.15729 26327300

[24] Burton G.J., Woods A.W., Jauniaux E. and Kingdom J. (2009) Rheological and physiological consequences of conversion of the maternal spiral arteries for uteroplacental blood flow during human pregnancy. Placenta 30, 473–482 10.1016/j.placenta.2009.02.009 19375795 PMC2697319

[25] Sun C., Groom K.M., Oyston C., Chamley L.W., Clark A.R. and James J.L. (2020) The placenta in fetal growth restriction: what is going wrong? Placenta 96, 10–18 10.1016/j.placenta.2020.05.003 32421528

[26] Proctor L.K., Toal M., Keating S., Chitayat D., Okun N., Windrim R.C. et al. (2009) Placental size and the prediction of severe early‐onset intrauterine growth restriction in women with low pregnancy‐associated plasma protein‐A. Ultrasound Obstetrics Gynecol.: Off. J. Int. Soc. Ultrasound Obstetrics Gynecol. 34, 274–282 10.1002/uog.7308 19672838

[27] Hafner E., Metzenbauer M., Höfinger D., Munkel M., Gassner R., Schuchter K. et al. (2003) Placental growth from the first to the second trimester of pregnancy in SGA-foetuses and pre-eclamptic pregnancies compared to normal foetuses. Placenta 24, 336–342 10.1053/plac.2002.0918 12657506

[28] Vachon-Marceau C., Demers S., Markey S., Okun N., Girard M., Kingdom J. et al. (2017) First-trimester placental thickness and the risk of preeclampsia or SGA. Placenta 57, 123–128 10.1016/j.placenta.2017.06.016 28864000

[29] Effendi M., Demers S., Giguere Y., Forest J., Brassard N., Girard M. et al. (2014) Association between first-trimester placental volume and birth weight. Placenta 35, 99–102 10.1016/j.placenta.2013.11.015 24345759

[30] Widdows K.L. (2009) Gestational related morphological abnormalities in placental villous trophoblast turnover in compromised pregnancies. http://bura.brunel.ac.uk/handle/2438/4444 [Accessed on 21 Dec 2023]

[31] Crocker I.P., Cooper S., Ong S.C. and Baker P.N. (2003) Differences in apoptotic susceptibility of cytotrophoblasts and syncytiotrophoblasts in normal pregnancy to those complicated with preeclampsia and intrauterine growth restriction. Am. J. Pathol. 162, 637–643 10.1016/S0002-9440(10)63857-6 12547721 PMC1851173

[32] Ishihara N., Matsuo H., Murakoshi H., Laoag-Fernandez J.B., Samoto T. and Maruo T. (2002) Increased apoptosis in the syncytiotrophoblast in human term placentas complicated by either preeclampsia or intrauterine growth retardation. Obstet. Gynecol. 186, 158–166 10.1067/mob.2002.119176 11810103

[33] Sibley C.P. (2009) Understanding placental nutrient transfer–why bother? new biomarkers of fetal growth J. Physiol. (Lond.) 587, 3431–3440 10.1113/jphysiol.2009.172403 19417095 PMC2742272

[34] Mahendran D., Donnai P., Glazier J.D., D'souza S.W., Boyd R.D. and Sibley C.P. (1993) Amino acid (system A) transporter activity in microvillous membrane vesicles from the placentas of appropriate and small for gestational age babies. Pediatr. Res. 34, 661–665 10.1203/00006450-199311000-00019 8284106

[35] Delmis J., Drazancic A., Ivanisevic M. and Suchanek E. (1992) Glucose, insulin, HGH and IGF-I levels in maternal serum, amniotic fluid and umbilical venous serum: A comparison between late normal pregnancy and pregnancies complicated with diabetes and fetal growth retardation. J. Perinat. Med. 20, 47–56 10.1515/jpme.1992.20.1.47 1608023

[36] Challis D.E., Pfarrer C.D., Ritchie J.W., Koren G. and Adamson S.L. (2000) Glucose metabolism is elevated and vascular resistance and maternofetal transfer is normal in perfused placental cotyledons from severely growth-restricted fetuses. Pediatr. Res. 47, 309–315 10.1203/00006450-200003000-00005 10709728

[37] Reynolds L.P., Vonnahme K.A., Lemley C.O., Redmer D.A., Grazul-Bilska A.T., Borowicz P.P. et al. (2013) Maternal stress and placental vascular function and remodeling. Curr. Vasc. Pharmacol. 11, 564–593 10.2174/1570161111311050003 24063377

[38] Pereira R.D., De Long N.E., Wang R.C., Yazdi F.T., Holloway A.C. and Raha S. (2015) Angiogenesis in the placenta: the role of reactive oxygen species signaling. Biomed. Res. Int. 2015, 10.1155/2015/814543 PMC4325211 25705690

[39] Alfaidy N., Hoffmann P., Boufettal H., Samouh N., Aboussaouira T., Benharouga M. et al. (2014) The multiple roles of EG-VEGF/PROK1 in normal and pathological placental angiogenesis. Biomed. Res. Int. 2014, 451906 10.1155/2014/451906 24955357 PMC4052057

[40] Baschat A.A. (2004) Pathophysiology of fetal growth restriction: implications for diagnosis and surveillance. Obstet. Gynecol. Surv. 59, 617–627 10.1097/01.OGX.0000133943.54530.76 15277896

[41] Herbert S.P. and Stainier D.Y. (2011) Molecular control of endothelial cell behaviour during blood vessel morphogenesis. Nat. Rev. Mol. Cell Biol. 12, 551–564 10.1038/nrm3176 21860391 PMC3319719

[42] Hogan B.L. and Kolodziej P.A. (2002) Molecular mechanisms of tubulogenesis. Nat. Rev. Genet. 3, 513–523 10.1038/nrg840 12094229

[43] Kaufmann P., Mayhew T.M. and Charnock-Jones D.S. (2004) Aspects of human fetoplacental vasculogenesis and angiogenesis. II. changes during normal pregnancy. Placenta 25, 114–126 10.1016/j.placenta.2003.10.009 14972444

[44] Barut F., Barut A., Gun B.D., Kandemir N.O., Harma M.I., Harma M. et al. (2010) Intrauterine growth restriction and placental angiogenesis. Diagon Pathol. 5, 24 10.1186/1746-1596-5-24 PMC2865442 20412591

[45] Vuorela P., Carpen O., Tulppala M. and Halmesmaki E. (2000) VEGF, its receptors and the tie receptors in recurrent miscarriage. Mol. Hum. Reprod. 6, 276–282 10.1093/molehr/6.3.276 10694277

[46] Gordon Z., Elad D., Almog R., Hazan Y., Jaffa A.J. and Eytan O. (2007) Anthropometry of fetal vasculature in the chorionic plate. J. Anat. 211, 698–706 10.1111/j.1469-7580.2007.00819.x 17973911 PMC2375851

[47] Sehgal A., Dahlstrom J.E., Chan Y., Allison B.J., Miller S.L. and Polglase G.R. (2019) Placental histopathology in preterm fetal growth restriction. J. Paediatr. Child Health 55, 582–587 10.1111/jpc.14251 30288833

[48] Junaid T.O., Brownbill P., Chalmers N., Johnstone E.D. and Aplin J.D. (2014) Fetoplacental vascular alterations associated with fetal growth restriction. Placenta 35, 808–815 10.1016/j.placenta.2014.07.013 25145956

[49] Junaid T.O., Bradley R.S., Lewis R.M., Aplin J.D. and Johnstone E.D. (2017) Whole organ vascular casting and microCT examination of the human placental vascular tree reveals novel alterations associated with pregnancy disease. Sci. Rep. 7, 4144–0 10.1038/s41598-017-04379-0 28646147 PMC5482861

[50] Sheibak N., Heidari Z., Mahmoudzadeh‐Sagheb H. and Narouei M. (2022) Reduced volumetric parameters of the placenta and extravillous trophoblastic cells in complicated pregnancies may lead to intrauterine growth restriction and small for gestational age birth. J. Obstet. Gynaecol. Res. 48, 1355–1363 10.1111/jog.15225 35293079

[51] Krebs C., Macara L.M., Leiser R., Bowman A.W., Greer I.A. and Kingdom J.C. (1996) Intrauterine growth restriction with absent end-diastolic flow velocity in the umbilical artery is associated with maldevelopment of the placental terminal villous tree. Obstet. Gynecol. 175, 1534–1542 10.1016/S0002-9378(96)70103-5 8987938

[52] Lu L., Kingdom J., Burton G.J. and Cindrova-Davies T. (2017) Placental stem villus arterial remodeling associated with reduced hydrogen sulfide synthesis contributes to human fetal growth restriction. Am. J. Pathol. 187, 908–920 10.1016/j.ajpath.2016.12.002 28157488 PMC5397715

[53] Boss A.L., Chamley L.W., Brooks A.E. and James J.L. (2023) Human placental vascular and perivascular cell heterogeneity differs between first trimester and term, and in pregnancies affected by fetal growth restriction. Mol. Hum. Reprod. 29, 1–14 10.1093/molehr/gaad041 38059603 PMC10746841

[54] Umapathy A., McCall A., Sun C., Boss A.L., Gamage T.K., Brooks A.E. et al. (2020) Mesenchymal stem/stromal cells from the placentae of growth restricted pregnancies are poor stimulators of angiogenesis. Stem Cell Rev. Rep. 16, 557–568 10.1007/s12015-020-09959-8 32080795

[55] Tun W.M., Yap C.H., Saw S.N., James J.L. and Clark A.R. (2019) Differences in placental capillary shear stress in fetal growth restriction may affect endothelial cell function and vascular network formation. Sci. Rep. 9, 1–10 10.1038/s41598-019-46151-6 31285454 PMC6614400

[56] Boss A.L., Chamley L.W., Brooks A.E. and James J.L. (2021) Differences in human placental mesenchymal stromal cells may impair vascular function in FGR. Reproduction 162, 319–330 10.1530/REP-21-0226 34397395

[57] Wang Y. (2010) Vascular biology of the placenta. 2, 1–98 10.4199/C00016ED1V01Y201008ISP009 21452443

[58] Ferrara N. (2004) Vascular endothelial growth factor: basic science and clinical progress. Endocr. Rev. 25, 581–611 10.1210/er.2003-0027 15294883

[59] Charnock-Jones D.S., Sharkey A.M., Boocock C.A., Ahmed A., Plevin R., Ferrara N. et al. (1994) Vascular endothelial growth factor receptor localization and activation in human trophoblast and choriocarcinoma cells. Biol. Reprod. 51, 524–530 10.1095/biolreprod51.3.524 7803624

[60] Haddad R. and Saldanha-Araujo F. (2014) Mechanisms of T-cell immunosuppression by mesenchymal stromal cells: what do we know so far? Biomed. Res. Int. 2014, 216806 10.1155/2014/216806 25025040 PMC4082893

[61] Luttun A. and Carmeliet P. (2003) Soluble VEGF receptor Flt1: the elusive preeclampsia factor discovered? J. Clin. Invest. 111, 600–602 10.1172/JCI18015 12618513 PMC151908

[62] Ahmed A., Li X.F., Dunk C., Whittle M.J., Rushton D.I. and Rollason T. (1995) Colocalisation of vascular endothelial growth factor and its flt-1 receptor in human placenta. Growth Factors 12, 235–243 10.3109/08977199509036883 8619929

[63] Carmeliet P., Ferreira V., Breier G. and Pollefeyt S. (1996) Abnormal blood vessel development and lethality in embryos lacking a single VEGF allele. Nature 380, 435 10.1038/380435a0 8602241

[64] Ferrara N. and Davis-Smyth T. (1997) The biology of vascular endothelial growth factor. Endocr. Rev. 18, 4–25 10.1210/edrv.18.1.0287 9034784

[65] Fong G., Rossant J., Gertsenstein M. and Breitman M.L. (1995) Role of the flt-1 receptor tyrosine kinase in regulating the assembly of vascular endothelium. Nature 376, 66–70 10.1038/376066a0 7596436

[66] Lyall F., Young A., Boswell F., Kingdom J. and Greer I.A. (1997) Placental expression of vascular endothelial growth factor in placentae from pregnancies complicated by pre-eclampsia and intrauterine growth restriction does not support placental hypoxia at delivery. Placenta 18, 269–276 10.1016/S0143-4004(97)80061-6 9179920

[67] Park J.E., Chen H.H., Winer J., Houck K.A. and Ferrara N. (1994) Placenta growth factor. potentiation of vascular endothelial growth factor bioactivity, in vitro and in vivo, and high affinity binding to flt-1 but not to flk-1/KDR. J. Biol. Chem. 269, 25646–25654 10.1016/S0021-9258(18)47298-5 7929268

[68] Kumazaki K., Nakayama M., Suehara N. and Wada Y. (2002) Expression of vascular endothelial growth factor, placental growth factor, and their receptors flt-1 and KDR in human placenta under pathologic conditions. Hum. Pathol. 33, 1069–1077 10.1053/hupa.2002.129420 12454810

[69] Dewerchin M. and Carmeliet P. (2014) Placental growth factor in cancer. Expert Opin. Ther. Targets 18, 1339–1354 10.1517/14728222.2014.948420 25297943

[70] Parchem J.G., Kanasaki K., Kanasaki M., Sugimoto H., Xie L., Hamano Y. et al. (2018) Loss of placental growth factor ameliorates maternal hypertension and preeclampsia in mice. J. Clin. Invest. 128, 5008–5017 10.1172/JCI99026 30179860 PMC6205389

[71] Kang M., Park S.J., Kim H.J., Lee J., Yu D.H., Bae K.B. et al. (2014) Gestational loss and growth restriction by angiogenic defects in placental growth factor transgenic mice. Arterioscler. Thromb. Vasc. Biol. 34, 2276–2282 10.1161/ATVBAHA.114.303693 25147341

[72] Khaliq A., Dunk C., Jiang J., Shams M., Li X.F., Acevedo C. et al. (1999) Hypoxia down-regulates placenta growth factor, whereas fetal growth restriction up-regulates placenta growth factor expression: Molecular evidence for “placental hyperoxia” in intrauterine growth restriction. Lab. Invest. 79, 151–170 10068204

[73] Joó J.G., Rigó J. Jr, Börzsönyi B., Demendi C. and Kornya L. (2017) Placental gene expression of the placental growth factor (PlGF) in intrauterine growth restriction. J. Matern. Fetal Neonatal Med. 30, 1471–1475 10.1080/14767058.2016.1219993 27483982

[74] Sheng J. and Xu Z. (2016) Three decades of research on angiogenin: a review and perspective. Acta Biochim. Biophys. Sin. 48, 399–410 10.1093/abbs/gmv131 26705141 PMC4888354

[75] König J., Weiss G., Rossi D., Wankhammer K., Reinisch A., Kinzer M. et al. (2014) Placental mesenchymal stromal cells derived from blood vessels or avascular tissues: what is the better choice to support endothelial cell function? Stem Cells Dev. 24, 115–131 10.1089/scd.2014.0115 PMC4273191 25244528

[76] Rajashekhar G., Loganath A., Roy A.C. and Wong Y.C. (2002) Expression and localization of angiogenin in placenta: enhanced levels at term over first trimester villi. Mol. Reprod. Dev. 62, 159–166 10.1002/mrd.10116 11984825

[77] Kishimoto K., Liu S., Tsuji T., Olson K.A. and Hu G. (2005) Endogenous angiogenin in endothelial cells is a general requirement for cell proliferation and angiogenesis. Oncogene 24, 445–456 10.1038/sj.onc.1208223 15558023

[78] Ahmed A. and Perkins J. (2000) Angiogenesis and intrauterine growth restriction. Baillieres Best Pract. Res. Clin. Obstet. Gynaecol. 14, 981–998 10.1053/beog.2000.0139 11141345

[79] Pavlov N., Hatzi E., Bassaglia Y., Frendo J., Evain-Brion D. and Badet J. (2003) Angiogenin distribution in human term placenta, and expression by cultured trophoblastic cells. Angiogenesis 6, 317–330 10.1023/B:AGEN.0000029412.95244.81 15166501 PMC1997312

[80] Rajashekhar G., Loganath A., Roy A.C. and Wong Y.C. (2003) Over‐expression and secretion of angiogenin in intrauterine growth retardation placenta. Mol. Reprod. Dev. 64, 397–404 10.1002/mrd.10229 12589651

[81] Shen H., Cavallero S., Estrada K.D., Sandovici I., Kumar S.R., Makita T. et al. (2015) Extracardiac control of embryonic cardiomyocyte proliferation and ventricular wall expansion. Cardiovasc. Res. 105, 271–278 10.1093/cvr/cvu269 25560321 PMC4366578

[82] Jones H.N., Olbrych S.K., Smith K.L., Cnota J.F., Habli M., Ramos-Gonzales O. et al. (2015) Hypoplastic left heart syndrome is associated with structural and vascular placental abnormalities and leptin dysregulation. Placenta 36, 1078–1086 10.1016/j.placenta.2015.08.003 26278057 PMC4609616

[83] Camm E.J., Botting K.J. and Sferruzzi-Perri A.N. (2018) Near to one’s heart: the intimate relationship between the placenta and fetal heart. Front Physiol. 9, 629 10.3389/fphys.2018.00629 29997513 PMC6029139

[84] Bulatovic I., Månsson-Broberg A., Sylvén C. and Grinnemo K. (2016) Human fetal cardiac progenitors: the role of stem cells and progenitors in the fetal and adult heart. Best Pract. Res. Clin. Obstet. Gynaecol. 31, 58–68 10.1016/j.bpobgyn.2015.08.008 26421632

[85] Savolainen S.M., Foley J.F. and Elmore S.A. (2009) Histology atlas of the developing mouse heart with emphasis on E11. 5 to E18. 5. Toxicol. Pathol. 37, 395–414 10.1177/0192623309335060 19359541 PMC2773446

[86] Dietrich A., Lombardo V.A., Veerkamp J., Priller F. and Abdelilah-Seyfried S. (2014) Blood flow and bmp signaling control endocardial chamber morphogenesis. Dev. Cell. 30, 367–377 10.1016/j.devcel.2014.06.020 25158852

[87] Männer J. (2009) The anatomy of cardiac looping: a step towards the understanding of the morphogenesis of several forms of congenital cardiac malformations. Clin. Anat. 22, 21–35 10.1002/ca.20652 18661581

[88] Buckingham M., Meilhac S. and Zaffran S. (2005) Building the mammalian heart from two sources of myocardial cells. Nat. Rev. Genet. 6, 826–835 10.1038/nrg1710 16304598

[89] Samsa L.A., Yang B. and Liu J. (2013) Embryonic cardiac chamber maturation: trabeculation, conduction, and cardiomyocyte proliferation. Am. J. Med. Genet. C Semin. Med. Genet. 163, 157–168 10.1002/ajmg.c.31366 PMC3723796 23720419

[90] Laflamme M.A. and Murry C.E. (2011) Heart regeneration. Nature 473, 326–335 10.1038/nature10147 21593865 PMC4091722

[91] Cavaliere T.A. (2016) From fetus to neonate: a sensational journey. Newborn Infant Nursing Rev. 16, 43–47 10.1053/j.nainr.2016.03.004

[92] Kiserud T., Ebbing C., Kessler J. and Rasmussen S. (2006) Fetal cardiac output, distribution to the placenta and impact of placental compromise. Ultrasound Obstet. Gynecol. 28, 126–136 10.1002/uog.2832 16826560

[93] Mayhew T.M., Gregson C. and Fagan D.G. (1999) Ventricular myocardium in control and growth-retarded human fetuses: growth in different tissue compartments and variation with fetal weight, gestational age, and ventricle size. Hum. Pathol. 30, 655–660 10.1016/S0046-8177(99)90090-4 10374773

[94] Crispi F., Miranda J. and Gratacós E. (2018) Long-term cardiovascular consequences of fetal growth restriction: biology, clinical implications, and opportunities for prevention of adult disease. Obstet. Gynecol. 218, S869–S879 10.1016/j.ajog.2017.12.012 29422215

[95] Sehgal A., Doctor T. and Menahem S. (2013) Cardiac function and arterial biophysical properties in small for gestational age infants: postnatal manifestations of fetal programming. J. Pediatr. 163, 1296–1300 10.1016/j.jpeds.2013.06.030 23896189

[96] Crispi F., Crovetto F. and Gratacos E. (2018) Intrauterine growth restriction and later cardiovascular function. Early Hum. Dev. 126, 23–27 10.1016/j.earlhumdev.2018.08.013 30206007

[97] Tsyvian P., Malkin K. and Wladimiroff J.W. (1995) Assessment of fetal left cardiac isovolumic relaxation time in appropriate and small-for-gestational-age fetuses. Ultrasound Med. Biol. 21, 739–743 10.1016/0301-5629(95)00016-K 8571461

[98] Corstius H.B., Zimanyi M.A., Maka N., Herath T., Thomas W., Van Der Laarse A. et al. (2005) Effect of intrauterine growth restriction on the number of cardiomyocytes in rat hearts. Pediatr. Res. 57, 796–800 10.1203/01.PDR.0000157726.65492.CD 15774830

[99] Bubb K.J., Cock M.L., Black M.J., Dodic M., Boon W., Parkington H.C. et al. (2007) Intrauterine growth restriction delays cardiomyocyte maturation and alters coronary artery function in the fetal sheep. J. Physiol. (Lond.) 578, 871–881 10.1113/jphysiol.2006.121160 17124269 PMC2151351

[100] Rizzo G., Capponi A., Rinaldo D., Arduini D. and Romanini C. (1995) Ventricular ejection force in growth‐retarded fetuses. Ultrasound Obstet. Gynecol. 5, 247–255 10.1046/j.1469-0705.1995.05040247.x 7600206

[101] Altın H., Karaarslan S., Karataş Z., Alp H., Şap F. and Baysal T. (2012) Evaluation of cardiac functions in term small for gestational age newborns with mild growth retardation: A serial conventional and tissue doppler imaging echocardiographic study. Early Hum. Dev. 88, 757–764 10.1016/j.earlhumdev.2012.04.003 22591553

[102] Fouzas S., Karatza A.A., Davlouros P.A., Chrysis D., Alexopoulos D., Mantagos S. et al. (2014) Neonatal cardiac dysfunction in intrauterine growth restriction. Pediatr. Res. 75, 651–657 10.1038/pr.2014.22 24522102

[103] Crispi F., Hernandez-Andrade E., Pelsers M.M., Plasencia W., Benavides-Serralde J.A., Eixarch E. et al. (2008) Cardiac dysfunction and cell damage across clinical stages of severity in growth-restricted fetuses. Obstet. Gynecol. 199, 254.e1–254.e8 10.1016/j.ajog.2008.06.056 18771973

[104] Girsén A., Ala‐Kopsala M., Mäkikallio K., Vuolteenaho O. and Räsänen J. (2007) Cardiovascular hemodynamics and umbilical artery n‐terminal peptide of proB‐type natriuretic peptide in human fetuses with growth restriction. Ultrasound Obstet. Gynecol. 29, 296–303 10.1002/uog.3934 17323307

[105] Vijlbrief D.C., van Bel F., Molenschot M.C., Benders M.J., Pistorius L.R., Kemperman H. et al. (2014) Early detection of prenatal cardiocirculatory compromise in small for gestational age infants. Neonatalogy 105, 256–262 10.1159/000357552 24556944

[106] Sehgal A., Allison B.J., Miller S.L. and Polglase G.R. (2023) Myocardial perfusion and function dichotomy in growth restricted preterm infants. J. Dev. Orig. Health Dis. 14, 302–310 10.1017/S2040174422000630 36408644

[107] Polglase G.R., Allison B.J., Coia E., Li A., Jenkin G., Malhotra A. et al. (2016) Altered cardiovascular function at birth in growth-restricted preterm lambs. Pediatr. Res. 80, 538–546 10.1038/pr.2016.104 27356081

[108] Sehgal A., Allison B.J., Gwini S.M., Miller S.L. and Polglase G.R. (2017) Cardiac morphology and function in preterm growth restricted infants: relevance for clinical sequelae. J. Pediatr. 188, 128.e2–134.e2 10.1016/j.jpeds.2017.05.076 28662946

[109] Sehgal A., Allison B.J., Gwini S.M., Menahem S., Miller S.L. and Polglase G.R. (2018) Vascular aging and cardiac maladaptation in growth-restricted preterm infants. J. Perinatol. 38, 92–97 10.1038/jp.2017.135 29120452

[110] Sehgal A., Allison B.J., Crispi F. and Menahem S. (2023) Influence of accelerated arterial aging in growth-restricted cohorts on adult-onset cardiovascular diseases. Am. J. Physiol. Heart Circ. Physiol. 325, H89–H105 10.1152/ajpheart.00134.2023 37204872

[111] Schorpp-Kistner M., Wang Z., Angel P. and Wagner E.F. (1999) JunB is essential for mammalian placentation. EMBO J. 18, 934–948 10.1093/emboj/18.4.934 10022836 PMC1171186

[112] Adams R.H., Porras A., Alonso G., Jones M., Vintersten K., Panelli S. et al. (2000) Essential role of p38α MAP kinase in placental but not embryonic cardiovascular development. Mol. Cell. 6, 109–116 10.1016/S1097-2765(05)00014-6 10949032

[113] Barak Y., Nelson M.C., Ong E.S., Jones Y.Z., Ruiz-Lozano P., Chien K.R. et al. (1999) PPARγ is required for placental, cardiac, and adipose tissue development. Mol. Cell. 4, 585–595 10.1016/S1097-2765(00)80209-9 10549290

[114] Radford B.N., Zhao X., Glazer T., Eaton M., Blackwell D., Mohammad S. et al. (2023) Defects in placental syncytiotrophoblast cells are a common cause of developmental heart disease. Nat. Commun. 14, 1174 10.1038/s41467-023-36740-5 36859534 PMC9978031

[115] Gessert S. and Kuhl M. (2010) The multiple phases and faces of wnt signaling during cardiac differentiation and development. Circ. Res. 107, 186–199 10.1161/CIRCRESAHA.110.221531 20651295

[116] Ahmad S.M. (2017) Conserved signaling mechanisms in drosophila heart development. Dev. Dyn. 246, 641–656 10.1002/dvdy.24530 28598558 PMC11546222

[117] Courtney J.A., Wilson R.L., Cnota J. and Jones H.N. (2021) Conditional mutation of Hand1 in the mouse placenta disrupts placental vascular development resulting in fetal loss in both early and late pregnancy. Int. J. Mol. Sci. 22, 9532 10.3390/ijms22179532 34502440 PMC8431056

[118] Knöfler M., Meinhardt G., Vasicek R., Husslein P. and Egarter C. (1998) Molecular cloning of the human Hand1 gene/cDNA and its tissue-restricted expression in cytotrophoblastic cells and heart. Gene 224, 77–86 10.1016/S0378-1119(98)00511-3 9931445

[119] Suryawanshi H., Morozov P., Straus A., Sahasrabudhe N., Max K.E., Garzia A. et al. (2018) A single-cell survey of the human first-trimester placenta and decidua. Sci. Adv. 4, eaau4788 10.1126/sciadv.aau4788 30402542 PMC6209386

[120] Barnes R.M., Firulli B.A., Conway S.J., Vincentz J.W. and Firulli A.B. (2010) Analysis of the Hand1 cell lineage reveals novel contributions to cardiovascular, neural crest, extra‐embryonic, and lateral mesoderm derivatives. Dev. Dyn. 239, 3086–3097 10.1002/dvdy.22428 20882677 PMC2965316

[121] Thattaliyath B.D., Livi C.B., Steinhelper M.E., Toney G.M. and Firulli A.B. (2002) HAND1 and HAND2 are expressed in the adult-rodent heart and are modulated during cardiac hypertrophy. Biochem. Biophys. Res. Commun. 297, 870–875 10.1016/S0006-291X(02)02297-0 12359233

[122] Breckenridge R.A., Zuberi Z., Gomes J., Orford R., Dupays L., Felkin L.E. et al. (2009) Overexpression of the transcription factor Hand1 causes predisposition towards arrhythmia in mice. J. Mol. Cell Cardiol. 47, 133–141 10.1016/j.yjmcc.2009.04.007 19376125

[123] Ottaviani G. and Buja L.M. (2022) Congenital heart disease: pathology, natural history, and interventions. Cardiovascular Pathology Congenital heart disease: Pathology, natural history, and interventions, pp. 223–264, Elsevier 10.1016/B978-0-12-822224-9.00011-6

[124] Firulli A.B., McFadden D.G., Lin Q., Srivastava D. and Olson E.N. (1998) Heart and extra-embryonic mesodermal defects in mouse embryos lacking the bHLH transcription factor Hand1. Nat. Genet. 18, 266–270 10.1038/ng0398-266 9500550

[125] Riley P., Anson-Cartwright L. and Cross J.C. (1998) The Hand1 bHLH transcription factor is essential for placentation and cardiac morphogenesis. Nat. Genet. 18, 271–275 10.1038/ng0398-271 9500551

[126] Liu H., Murthi P., Qin S., Kusuma G.D., Borg A.J., Knöfler M. et al. (2014) A novel combination of homeobox genes is expressed in mesenchymal chorionic stem/stromal cells in first trimester and term pregnancies. Reprod. Sci. 21, 1382–1394 10.1177/1933719114526471 24692208 PMC4212331

[127] Pathirage N.A., Cocquebert M., Sadovsky Y., Abumaree M., Manuelpillai U., Borg A. et al. (2013) Homeobox gene transforming growth factor β-induced factor-1 (TGIF-1) is a regulator of villous trophoblast differentiation and its expression is increased in human idiopathic fetal growth restriction. Mol. Hum. Reprod. 19, 665–675 10.1093/molehr/gat042 23761267

[128] Gunatillake T., Yong H.E., Dunk C., Keogh R.J., Borg A.J., Cartwright J.E. et al. (2016) Homeobox gene TGIF-1 is increased in placental endothelial cells of human fetal growth restriction. Reproduction 152, 457–465 10.1530/REP-16-0068 27539603

[129] Chui A., Tay C., Cocquebert M., Sheehan P., Pathirage N.A., Donath S. et al. (2012) Homeobox gene distal-less 3 is a regulator of villous cytotrophoblast differentiation and its expression is increased in human idiopathic foetal growth restriction. J. Mol. Med. (Berl.) 90, 273–284 10.1007/s00109-011-0836-1 22113468

[130] Murthi P., Hiden U., Rajaraman G., Liu H., Borg A.J., Coombes F. et al. (2008) Novel homeobox genes are differentially expressed in placental microvascular endothelial cells compared with macrovascular cells. Placenta 29, 624–630 10.1016/j.placenta.2008.04.006 18514308

[131] Murthi P., So M., Gude N.M., Doherty V.L., Brennecke S.P. and Kalionis B. (2007) Homeobox genes are differentially expressed in macrovascular human umbilical vein endothelial cells and microvascular placental endothelial cells. Placenta 28, 219–223 10.1016/j.placenta.2006.02.012 16647116

[132] Murthi P., Doherty V., Said J., Donath S., Brennecke S.P. and Kalionis B. (2006) Homeobox gene HLX1 expression is decreased in idiopathic human fetal growth restriction. Am. J. Pathol. 168, 511–518 10.2353/ajpath.2006.050637 16436665 PMC1606485

[133] Murthi P., Doherty V.L., Said J.M., Donath S., Brennecke S.P. and Kalionis B. (2006) Homeobox gene ESX1L expression is decreased in human pre-term idiopathic fetal growth restriction. Mol. Hum. Reprod. 12, 335–340 10.1093/molehr/gal037 16613891

[134] Maslen C.L. (2018) Recent advances in placenta–heart interactions. Front Physiol. 9, 735 10.3389/fphys.2018.00735 29962966 PMC6010578

[135] Shaut C.A., Keene D.R., Sorensen L.K., Li D.Y. and Stadler H.S. (2008) HOXA13 is essential for placental vascular patterning and labyrinth endothelial specification. PLos Genet. 4, e1000073 10.1371/journal.pgen.1000073 18483557 PMC2367452

[136] Cecchetto A., Rampazzo A., Angelini A., Bianco L.D., Padalino M., Stellin G. et al. (2010) From molecular mechanisms of cardiac development to genetic substrate of congenital heart diseases. Future Cardiol. 6, 373–393 10.2217/fca.10.10 20462343

[137] Roux M. and Zaffran S. (2016) Hox genes in cardiovascular development and diseases. J. Dev. Biol. 4, 14 10.3390/jdb4020014 29615581 PMC5831787

[138] Miksiunas R., Mobasheri A. and Bironaite D. (2020) Homeobox genes and homeodomain proteins: new insights into cardiac development, degeneration and regeneration. Adv. Exp. Med. Biol. 1212, 155–178 10.1007/5584_2019_349 30945165

